# Disrupted intrathalamic and thalamocortical structural covariance networks in posttraumatic stress disorder

**DOI:** 10.1162/NETN.a.535

**Published:** 2026-03-20

**Authors:** Nick Steele, Ahmed Hussain, Delin Sun, C. Lexi Baird, Courtney Russell, Neda Jahanshad, Lauren E. Salminen, Miranda Olff, Jessie L. Frijling, Dick J. Veltman, Saskia B. J. Koch, Laura Nawijn, Mirjam van Zuiden, Li Wang, Ye Zhu, Gen Li, Dan J. Stein, Jonathan Ipser, Sheri Koopowitz, Yuval Neria, Xi Zhu, Orren Ravid, Sigal Zilcha-Mano, Amit Lazarov, Benjamin Suarez-Jimenez, Ashley A. Huggins, Jennifer Stevens, Kerry Ressler, Tanja Jovanovic, Sanne J. H. van Rooij, Negar Fani, Emily L. Dennis, David F. Tate, David X. Cifu, William C. Walker, Elisabeth A. Wilde, Ivan Rektor, Pavel Říha, Milissa L. Kaufman, Lauren A. M. Lebois, Justin T. Baker, Anthony King, Israel Liberzon, Mike Angstadt, Nicholas D. Davenport, Seth G. Disner, Scott R. Sponheim, Thomas Straube, David Hofmann, Guang Ming Lu, Rongfeng Qi, Amit Etkin, Adi Maron-Katz, Xin Wang, Austin Kunch, Hong Xie, Yann Quidé, Wissam El-Hage, Shmuel Lissek, Hannah Berg, Steven E. Bruce, Josh Cisler, Marisa Ross, Ryan Herringa, Daniel W. Grupe, Jack B. Nitschke, Richard J. Davidson, Christine Larson, Terri A. deRoon-Cassini, Carissa W. Tomas, Jacklynn M. Fitzgerald, Brandee Feola, Jennifer U. Blackford, Bunmi O. Olatunji, Geoffrey May, Steven M. Nelson, Evan M. Gordon, Chadi G. Abdallah, Ruth Lanius, Maria Densmore, Jean Théberge, Richard W. J. Neufeld, Paul M. Thompson, Rajendra A. Morey

**Affiliations:** Brain Imaging and Analysis Center, Duke University, Durham, NC, USA; Department of Veteran Affairs Mid-Atlantic Mental Illness Research, Education and Clinical Center, Durham, NC, USA; Department of Psychiatry and Behavioral Sciences, School of Medicine, Duke University, Durham, NC, USA; Department of Psychology, Pennsylvania State University, State College, PA, USA; Imaging Genetics Center, Mark and Mary Stevens Neuroimaging & Informatics Institute, Keck School of Medicine of USC, Marina del Rey, CA, USA; Department of Psychiatry, Amsterdam UMC, Amsterdam, The Netherlands; ARQ National Psychotrauma Centre, Diemen, The Netherlands; De Viersprong mental health specialist in personality disorders, family and behavior, Amsterdam, The Netherlands; Amsterdam Neuroscience, Amsterdam, The Netherlands; Donders Institute for Brain, Cognition and Behavior, Centre for Cognitive Neuroimaging, Radboud University Nijmegen, Nijmegen, The Netherlands; Department of Clinical Psychology, Utrecht University, Utrecht, The Netherlands; Laboratory for Traumatic Stress Studies, Chinese Academy of Sciences Key Laboratory of Mental Health, Institute of Psychology, Chinese Academy of Sciences, Beijing, China; Department of Psychology, University of Chinese Academy of Sciences, Beijing, China; SA MRC Unit on Risk & Resilience in Mental Disorders, Department of Psychiatry and Neuroscience Institute, University of Cape Town, Cape Town, South Africa; Department of Psychiatry, Columbia University Medical Center, New York, NY, USA; New York State Psychiatric Institute, New York, NY, USA; University of Haifa, Haifa, Israel; School of Psychological Sciences, Tel-Aviv University, Tel Aviv, Israel; Department of Neuroscience, University of Rochester Medical Center, Rochester, NY, USA; Department of Psychology, University of Arizona, Tucson, AZ, USA; Department of Psychiatry and Behavioral Sciences, Emory University School of Medicine, Atlanta, GA, USA; Division of Depression and Anxiety Disorders, McLean Hospital, Belmont, MA, USA; Department of Psychiatry, Harvard Medical School, Boston, MA, USA; Department of Psychiatry and Behavioral Neuroscience, Wayne State University School of Medicine, Detroit, MI, USA; Department of Neurology, TBI and Concussion Center, University of Utah School of Medicine, Salt Lake City, UT, USA; George E. Wahlen Veterans Affairs Medical Center, Salt Lake City, UT, USA; Department of Physical Medicine and Rehabilitation, Virginia Commonwealth University, Richmond, VA, USA; Central Virginia Veterans Healthcare Systems, Richmond, VA, USA; H. Ben Taub Department of Physical Medicine and Rehabilitation, Baylor College of Medicine, Houston, TX, USA; CEITEC-Central European Institute of Technology, Multimodal and Functional Neuroimaging Research Group, Masaryk University, Brno, Czech Republic; First Department of Neurology, St. Anne's University Hospital and Faculty of Medicine, Masaryk University, Brno, Czech Republic; Division of Women's Mental Health, McLean Hospital, Belmont, MA, USA; Institute for Technology in Psychiatry, McLean Hospital, Belmont, MA, USA; Department of Psychiatry, University of Michigan, Ann Arbor, MI, USA; Department of Psychiatry, Texas A&M University, Bryan, TX, USA; Minneapolis VA Health Care System, Minneapolis, MN, USA; Department of Psychiatry, University of Minnesota, Minneapolis, MN, USA; Institute of Medical Psychology and Systems Neuroscience, University of Münster, Münster, Germany; Department of Medical Imaging, Jinling Hospital, Medical School of Nanjing University, Nanjing, China; Department of Psychiatry and Behavioral Sciences, Stanford University, Stanford, Palo Alto, CA, USA; VA Palo Alto Health Care System, Palo Alto, CA, USA; Department of Psychiatry, University of Toledo, Toledo, OH, USA; School of Psychology, University of New South Wales, Sydney, NSW, Australia; Neuroscience Research Australia, Randwick, NSW, Australia; UMR1253, iBraiN, Université de Tours, Inserm, Tours, France; Department of Psychology, University of Minnesota, Minneapolis, MN, USA; University of Missouri-St. Louis, Department of Psychological Sciences, Center for Trauma Recovery, St. Louis, MO, USA; Department of Psychiatry, University of Texas at Austin, Austin, TX, USA; Northwestern Neighborhood and Network Initiative, Northwestern University Institute for Policy Research, Evanston, IL, USA; School of Medicine and Public Health, University of Wisconsin Madison, Madison, WI, USA; Center for Healthy Minds, University of Wisconsin-Madison, Madison, WI, USA; Department of Psychiatry, University of Wisconsin-Madison, Madison, WI, USA; Department of Psychology, University of Wisconsin-Madison, Madison, WI, USA; Department of Psychology, University of Wisconsin-Milwaukee, Milwaukee, WI, USA; Division of Trauma and Acute Care Surgery, Department of Surgery, Medical College of Wisconsin, WI, USA; Comprehensive Injury Center, Medical College of Wisconsin, WI, USA; Department of Epidemiology, Institute of Health and Equity, Medical College of Wisconsin, WI, USA; Department of Psychology, Marquette University, Milwaukee, WI, USA; Department of Psychiatry and Behavioral Sciences, Vanderbilt University Medical Center, TN, USA; University of Nebraska Medical Center, Munroe-Meyer Institute, Omaha, NE, USA; Department of Psychology, Vanderbilt University, TN, USA; Center for Vital Longevity, School of Behavioral and Brain Sciences, University of Texas at Dallas, Dallas, TX, USA; Department of Psychology and Neuroscience, Baylor University, Waco, TX, USA; Veterans Integrated Service Network-17 Center of Excellence for Research on Returning War Veterans, Waco, TX, USA; Department of Psychiatry and Behavioral Science, Texas A&M University Health Science Center, Bryan, TX, USA; Department of Radiology, Washington University School of Medicine, St. Louis, MO, USA; Department of Psychiatry, Baylor College of Medicine, Houston, TX, USA; Department of Psychiatry, Yale University School of Medicine, New Haven, CT, USA; Department of Neuroscience, Western University, London, ON, Canada; Department of Psychiatry, Western University, London, ON, Canada

**Keywords:** Thalamus, Thalamocortical connections, Structural covariance, Graph theory, PTSD, Psychopathology

## Abstract

The thalamus is a heterogeneous structure crucial for corticocortical communication, affective–perceptual integration, motor preparation, and memory-related functions. Posttraumatic stress disorder (PTSD) is characterized by various symptoms that likely relate to thalamic functions. Group-level and individual differential structural covariance (SC) analyses were conducted on intrathalamic, thalamocortical, and thalamosubcortical volumetric networks by segmenting structural MRI data from 2,784 subjects (PTSD *n* = 1,306; controls *n* = 1,478) into 25 thalamic nuclei per hemisphere. We found that PTSD was associated with stronger intrathalamic and thalamocortical network strength and stronger SC between the limbic thalamus and the somatomotor and auditory thalamus. PTSD severity was related to specific regional alterations in the intrathalamic network involving the lateral pulvinar. Comorbid depression severity positively correlated with global intrathalamic alterations, while avoidance symptoms positively correlated with global thalamosubcortical alterations. Hyperarousal symptoms related to altered SC in the thalamocortical network between the reuniens, central medial, paratenial, centromedian, and limitans-suprageniculate nuclei and lateral cortical regions spanning the occipital, temporal, and orbitofrontal cortices. Differential associations between avoidance, hyperarousal, and comorbid depression symptoms and thalamic SC in PTSD suggest that specific thalamic covariance patterns may be involved in unique facets of PTSD symptomatology.

## Author Summary

The thalamus has been increasingly implicated in psychiatric disorders, including posttraumatic stress disorder (PTSD). However, most studies on PTSD have analyzed the thalamus as a whole, overlooking the distinct functional roles of its nuclei in cognitive, affective, and sensorimotor processes. This study examined structural covariance (SC) patterns of individual thalamic nuclei and found that PTSD diagnosis was related to differences in thalamic SC. While traditional studies of SC occur at the group level, we additionally conducted a subject-level SC analysis, which showed that distinct aspects of thalamic SC were linked to different PTSD symptoms. Comorbid depression was related to intrathalamic alterations, avoidance symptoms to thalamosubcortical alterations, and hyperarousal symptoms to thalamocortical alterations. This work should inform future research examining thalamic nuclei in relation to PTSD and other psychopathologies.

## INTRODUCTION

With a near 4% global prevalence rate (Koenen et al., 2017), posttraumatic stress disorder (PTSD) imposes both societal burdens and dramatic challenges to afflicted individuals. PTSD is characterized by intrusive memories and re-experiencing of a traumatic event, avoidance of internal and external reminders of the trauma, negative mood and cognition, and hyperarousal symptoms (American Psychiatric Association, 2022).

The thalamus is a heterogeneous structure composed of dozens of functionally distinct regions. Thalamic nuclei provide crucial inputs to the rest of the brain and facilitate corticocortical communication, relay and modulate sensorimotor information across primary and association perceptual cortices, and integrate affective information with ongoing behavioral goals across the higher-order cortex (Murray & Guillery, 2006; Sherman, 2016). Many of these thalamic functions mediate neurobehavioral processes that are likely involved in symptoms of PTSD. Identifying which thalamic nuclei may be altered in PTSD is of crucial importance for deepening our understanding of how and why PTSD symptoms arise. The functional heterogeneity of the thalamus necessitates examination of specific thalamic nuclei, while its broad connectivity profile highlights the need to examine thalamic connectivity with the rest of the brain.

Prior research found altered whole thalamus and thalamic nuclei volume in PTSD (Casteen et al., 2022; Huffman et al., 2023; Lopez-Larson et al., 2013; Shucard et al., 2012), predominantly among sensorimotor nuclei (Casteen et al., 2022; Xie et al., 2022; Yang et al., 2023) and the mediodorsal nucleus (Xie et al., 2022; Yang et al., 2023). Altered thalamic function both at rest and during recall of traumatic events (S. J. Kim et al., 2007; Lanius et al., 2001) and altered whole thalamus functional connectivity to the hippocampus, ventromedial prefrontal cortex, insula, and sensorimotor cortices (Harricharan et al., 2020; Jeon et al., 2020; Marlatte et al., 2022; Yin et al., 2011) have been consistently reported in PTSD. Yet, investigations of alterations to specific thalamic nuclei in PTSD are sparse, with no published studies on disrupted patterns of volumetric covariation in PTSD.

We sought to understand how thalamic nuclei are affected in PTSD by investigating differences in intrathalamic, thalamocortical, and thalamosubcortical network architecture by applying a structural covariance (SC) analysis. SC analysis characterizes covarying patterns of regional brain morphometry within a sample. Typical morphometric features include volume, cortical thickness, and surface area. Examining their covariance structure may offer unique insights about the formation of functionally meaningful networks within a sample. SC has been linked to both functional and direct structural (e.g., white matter) connectivity (Damoiseaux & Greicius, 2009; Paquola et al., 2018; Yee et al., 2018). Between-group differences in regional volumetric SC can thus help to uncover altered brain network dynamics, such as how brain regions share and integrate information. Bidirectional thalamocortical projections, interconnections with brainstem and subcortical sites, and direct intrathalamic connections between thalamic nuclei play an important role in the processing and integration of neural information (Guillery et al., 1998; Murray & Guillery, 2006; Sherman, 2016). Examining intrathalamic, thalamocortical, and thalamosubcortical SC differences (SC_diff_) in PTSD is needed for comprehensive insight into how systems involving thalamic nuclei may be impacted by the disorder. We hypothesized that global network properties of the thalamus would be abnormally altered in PTSD. We further hypothesized, given consistent reports of disrupted thalamocortical functional connectivity, that there would be detectable differences in the SC of thalamic nuclei with limbic regions and sensorimotor regions in PTSD.

Structural brain MRI scans from a total of 2,784 subjects, acquired at 28 worldwide sites, were shared for a secondary analysis with the ENIGMA (Enhancing Neuroimaging Genetics through Meta-Analysis)-PGC PTSD working group. The thalamus was segmented into 25 nuclei per hemisphere, including the mediodorsal medial (MDm), mediodorsal lateral (MDl), central medial (CeM), reuniens (Re), anteroventral (AV), paratenial (Pt), centromedian (CM), parafascicular (Pf), central lateral (CL), paracentral (Pc), lateral dorsal (LD), lateral posterior (LP), lateral geniculate nucleus (LGN), medial geniculate nucleus (MGN), limitans-suprageniculate (LSg), inferior pulvinar (PuI), lateral pulvinar (PuL), anterior pulvinar (PuA), medial pulvinar (PuM), ventral posterolateral (VPL), ventral medial (VM), ventral lateral anterior (VLa), ventral lateral posterior (VLp), ventral anterior (VA), and ventral anterior magnocellular (VAmc) nuclei.

**Figure 1.**
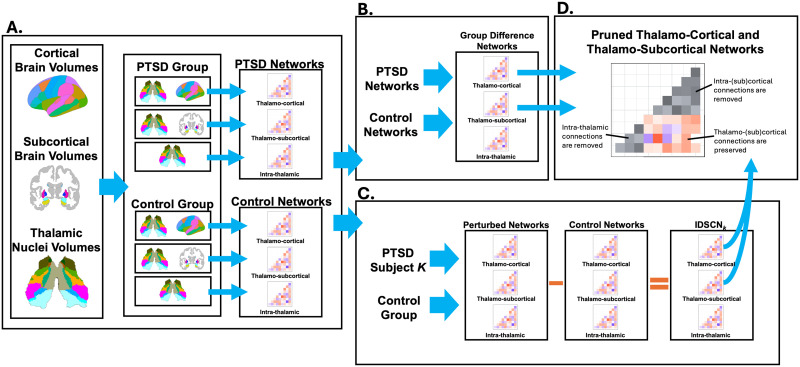
(A) Thalamic nuclei, cortical, and subcortical brain volumes were used to construct intrathalamic, thalamocortical, and thalamosubcortical networks for PTSD and control subjects. (B) A group-level analysis was performed by comparing the difference between the PTSD and control network to a null distribution generated by randomly shuffling diagnostic labels. (C) IDSCNs were created by adding a single PTSD subject into the control group to create a perturbed network then calculating the difference between the perturbed and control network to generate an IDSCN for the k^th^ PTSD subject. (D) Intrathalamic and intra(sub)cortical edges were removed from the thalamocortical and thalamosubcortical networks to isolate thalamo(sub)cortical connections.

Weighted intrathalamic (*n* = 2,784), thalamocortical (*n* = 2,343), and thalamosubcortical (*n* = 1,874) network graphs were generated using two distinct analytic methods: (a) group-level SC networks (SCNs) for PTSD and control groups, and (b) individual differential SCNs (IDSCNs). Weighted graphs were constructed by applying group-independent thresholds to correlation matrices for subjects with PTSD and trauma-exposed control subjects (Figure 1A). Global, nodal, and edge strengths were calculated for intrathalamic, thalamocortical, and thalamosubcortical networks and tested for between-group SC_diff_ (Figure 1B) for the unthresholded graph and across graph thresholds by comparing the observed group difference to a null distribution via permutation tests.

IDSCNs were generated using the *Template Network Perturbation* method (Figure 1C; Liu et al., 2021). PTSD severity, *Diagnostic and Statistical Manual of Mental Disorders*-*5*-defined PTSD symptom cluster severity (re-experiencing, avoidance, negative mood/cognition, and hyperarousal symptoms), and comorbid depression severity were tested for correlations with the number of altered edges and edge strengths across the subject-level IDSCNs. For both IDSCNs and group-level network analyses, thalamocortical and thalamosubcortical network connections were isolated by removing intrathalamic and intracortical/intrasubcortical edges from the networks (Figure 1D).

## RESULTS

### Validation of Thalamocortical SC Patterns

**Figure 2.**
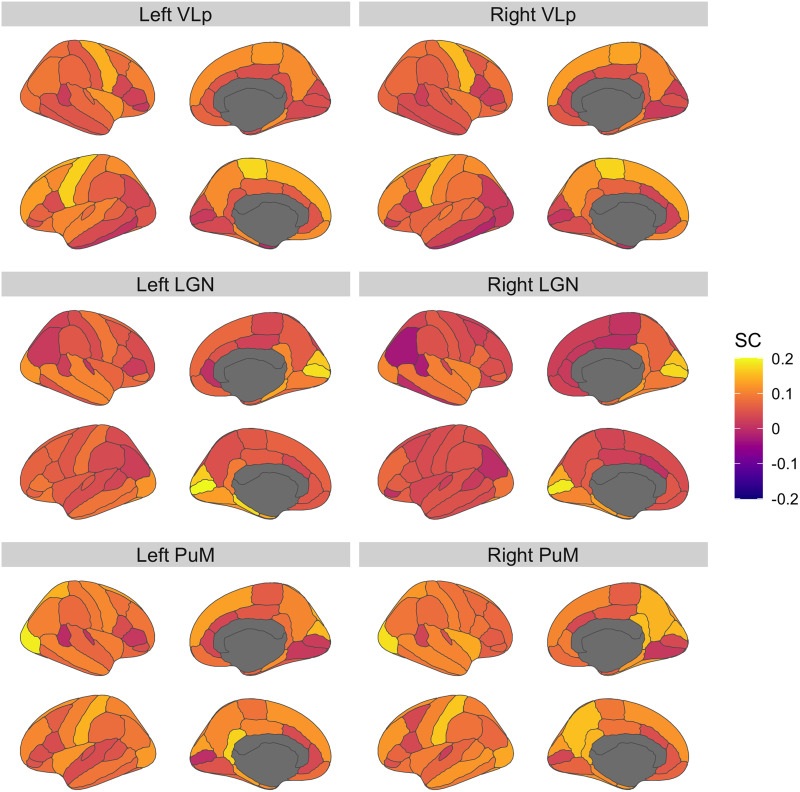
SC patterns (Pearson's correlation coefficient) of select thalamic nuclei with the cortex among control subjects. Covariance patterns closely align with prior reports of structural connections of these nuclei.

We first attempted to recapitulate established connectivity patterns of thalamocortical structures as a validation step. The SC patterns of thalamocortical connections were examined within subjects in the control group. Patterns of SC closely aligned with previous reports of structural connections between thalamic nuclei and cortical regions. For instance, the VLp motor nucleus displayed the highest SC with the motor cortex (Yee et al., 2024), the visual LGN displayed the highest SC with the occipital cortex (Andrews et al., 1997; Yee et al., 2024), and the heteromodal PuM displayed the highest SC with both somatosensory and visual regions (Benevento & Davis, 1977; Impieri et al., 2018; Figure 2).

### Altered Intrathalamic and Thalamocortical Global and Nodal Strength in PTSD

#### Graph strength.

Graph strength was compared between the PTSD and control groups. All *p* values reported in the text refer to the unthresholded graph. The intrathalamic network displayed higher graph strength (*p* = 0.013) in PTSD compared with controls. Group differences in graph strength remained stable across all thresholds (all *p* < 0.05).

The thalamocortical network displayed significantly higher graph strength (*p* = 0.040) in PTSD compared with controls. Group differences in graph strength remained stable across all thresholds (all *p* < 0.05).

No significant differences in graph strength were observed for the thalamosubcortical network in PTSD compared with controls (*p* > 0.05). Results across all graph thresholds for each network are available in Supporting Information Table S3.

#### Node centrality.

Node strength centrality was computed for thalamic nuclei in each network. In the intrathalamic network, 12 of the 50 thalamic nuclei displayed significantly higher centrality in PTSD, including the bilateral LSg, CM, and Re nuclei and the left MGN, VA, VPL, MDm, MDl, and CeM nuclei (all *p*_FDR_ < 0.05). These effects remained significant across graph thresholds.

No significant differences in node strength centrality were observed for the thalamocortical or thalamosubcortical networks in PTSD compared with controls (all *p*_FDR_ > 0.05). Centrality results for the unthresholded graph of each network can be seen in Table 1.

**Table 1.** Node strength centrality in the unthresholded graph of each network

Thalamic nucleus	PTSD	Controls	SC_diff_	*p*_FDR_	PTSD	Controls	SC_diff_	*p*_FDR_	PTSD	Controls	SC_diff_	*p*_FDR_
Left PuI	12.139	10.945	1.194	0.304	5.861	3.072	2.789	0.101	1.519	1.401	0.118	0.948
Right PuI	12.3	10.653	1.648	0.174	5.384	3.755	1.63	0.372	1.674	1.429	0.245	0.948
Left PuL	11.036	8.826	2.21	0.081	3.026	2.499	0.527	0.711	0.725	1.044	−0.319	0.948
Right PuL	11.299	9.415	1.884	0.113	4.601	1.712	2.889	0.073	1.009	1.195	−0.186	0.948
Left PuA	18.179	16.28	1.899	0.092	6.815	5.124	1.69	0.372	2.146	2.42	−0.274	0.948
Right PuA	18.419	16.258	2.161	0.064	7.958	6.008	1.95	0.349	2.229	2.519	−0.289	0.948
Left PuM	17.761	16.078	1.683	0.131	7.265	5.293	1.971	0.349	2.143	2.09	0.053	0.948
Right PuM	18.485	16.554	1.931	0.092	7.914	5.919	1.994	0.349	2.327	2.219	0.109	0.948
Left MGN	**14.14**	**11.603**	**2.537**	**0.049**	4.604	3.649	0.955	0.608	1.629	1.683	−0.054	0.948
Right MGN	14.052	12.117	1.935	0.113	3.998	3.765	0.233	0.878	1.709	1.789	−0.08	0.948
Left LSg	**11.279**	**8.755**	**2.525**	**0.049**	3.516	2.086	1.43	0.372	1.158	1.369	−0.211	0.948
Right LSg	**12.76**	**9.646**	**3.115**	**0.049**	3.171	2.222	0.949	0.545	1.219	1.406	−0.187	0.948
Left LGN	14.089	12.586	1.504	0.178	6.798	4.837	1.961	0.349	2.266	1.739	0.527	0.948
Right LGN	13.45	12.483	0.967	0.384	5.293	3.792	1.501	0.38	2.039	1.891	0.148	0.948
Left VPL	21.156	18.734	2.422	0.035	4.481	4.019	0.462	0.794	2.052	1.996	0.056	0.948
Right VPL	20.198	18.869	1.33	0.161	4.04	3.438	0.602	0.711	2.16	2.16	0	0.999
Left VLa	21.673	20.187	1.486	0.106	5.187	4.251	0.936	0.611	2.299	2.506	−0.207	0.948
Right VLa	21.294	20.229	1.065	0.223	7.057	4.082	2.975	0.101	2.401	2.644	−0.243	0.948
Left VLp	22.181	20.794	1.387	0.113	5.411	4.751	0.66	0.711	2.29	2.451	−0.161	0.948
Right VLp	22.159	20.932	1.227	0.162	6.494	4.312	2.182	0.32	2.412	2.493	−0.08	0.948
Left VM	18.104	16.205	1.899	0.081	2.117	2.009	0.108	0.934	1.657	1.647	0.01	0.997
Right VM	18.093	16.558	1.535	0.131	2.412	2.124	0.288	0.838	1.958	1.867	0.091	0.948
Left VA	**20.151**	**17.926**	**2.226**	**0.049**	5.167	4.677	0.49	0.791	2.278	2.548	−0.27	0.948
Right VA	19.96	18.687	1.273	0.176	7.859	4.242	3.617	0.055	2.546	2.901	−0.355	0.948
Left VAmc	21.224	19.783	1.441	0.113	4.886	3.288	1.598	0.372	2.422	2.509	−0.087	0.948
Right VAmc	21.38	20.176	1.204	0.17	6.276	3.044	3.232	0.073	2.638	2.909	−0.271	0.948
Left LP	13.911	14.549	−0.639	0.53	6.832	7.493	−0.661	0.711	1.82	2.166	−0.346	0.948
Right LP	**14.48**	**13.768**	**0.712**	**0.49**	6.124	5.527	0.597	0.711	1.811	1.795	0.016	0.997
Left LD	10.447	12.023	−1.576	0.174	5.752	3.758	1.994	0.32	1.439	1.804	−0.366	0.948
Right LD	10.977	10.323	0.653	0.53	4.324	3.083	1.241	0.437	1.368	1.359	0.008	0.997
Left CL	13.701	14.243	−0.542	0.57	3.077	3.369	−0.292	0.838	1.361	1.863	−0.502	0.948
Right CL	13.757	13.22	0.537	0.57	3.259	2.986	0.273	0.849	1.457	1.549	−0.092	0.948
Left Pc	18.218	17.087	1.131	0.233	4.354	2.873	1.482	0.38	2.037	2.358	−0.32	0.948
Right Pc	17.34	16.339	1.001	0.32	5.285	3.644	1.641	0.372	2.099	2.524	−0.425	0.948
Left Pf	18.072	16.762	1.309	0.179	3.848	3.804	0.044	0.969	2.141	2.395	−0.254	0.948
Right Pf	18.108	17.217	0.892	0.371	5.629	3.789	1.84	0.354	2.518	2.432	0.086	0.948
Left CM	**22.153**	**20.13**	**2.023**	**0.049**	5.304	3.996	1.308	0.468	2.604	2.496	0.108	0.948
Right CM	**21.874**	**19.853**	**2.021**	**0.049**	5.78	4.022	1.758	0.372	2.857	2.653	0.204	0.948
Left MDl	**16.115**	**13.833**	**2.282**	**0.049**	6.889	5.699	1.19	0.509	2.132	2.194	−0.062	0.948
Right MDl	15.755	13.904	1.851	0.112	7.571	5.778	1.794	0.372	2.17	2.24	−0.07	0.948
Left MDm	**18.027**	**15.749**	**2.278**	**0.049**	6.768	5.486	1.283	0.468	2.638	2.509	0.129	0.948
Right MDm	17.258	15.791	1.468	0.161	7.508	6.397	1.111	0.545	2.475	2.579	−0.104	0.948
Left Re	**16.976**	**14.534**	**2.442**	**0.049**	6.532	3.478	3.053	0.073	2.877	2.541	0.336	0.948
Right Re	**17.381**	**14.494**	**2.887**	**0.035**	6.68	3.096	3.584	0.055	2.973	2.577	0.396	0.948
Left CeM	**19.49**	**17.369**	**2.121**	**0.049**	5.053	2.889	2.164	0.253	2.647	2.715	−0.068	0.948
Right CeM	19.657	17.704	1.952	0.071	5.935	2.886	3.049	0.073	2.615	2.91	−0.295	0.948
Left Pt	21.121	19.703	1.418	0.144	4.9	3.952	0.948	0.608	1.855	2.228	−0.372	0.948
Right Pt	23.101	21.652	1.448	0.113	5.862	4.36	1.502	0.387	2.539	2.729	−0.19	0.948
Left AV	17.107	16.264	0.843	0.384	4.479	3.77	0.709	0.711	2.083	2.525	−0.443	0.948
Right AV	16.445	15.562	0.883	0.384	5.45	3.092	2.358	0.219	1.834	2.381	−0.547	0.948

Statistically significant values are **bolded**.

### Greater Intrathalamic Edge Strengths in PTSD

**Figure 3.**
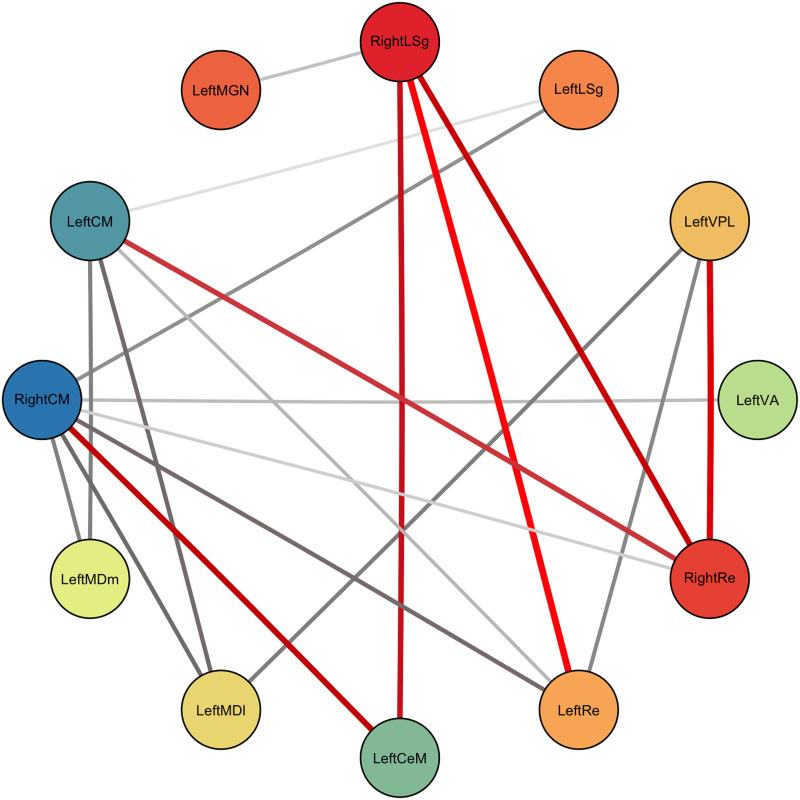
Network graph displaying significantly altered intrathalamic network edges among nuclei with significantly altered centrality in PTSD. Network edges with larger SC_diff_ appear redder and thicker (edge weights have been cubically scaled to emphasize differences). Nodes with a larger between-group strength difference appear redder/warmer in color.

Intrathalamic network edges between the 12 nuclei with significantly altered centrality in PTSD were analyzed. After correction for multiple comparisons, 19 edges showed significantly greater strength in PTSD compared with controls (Figure 3). Edges with the greatest SC_diff_ were between the Re and CeM nuclei and the LSg, VPL, and CM nuclei (Supporting Information Table S4). No significant results emerged when expanding the analysis to include all 1,225 edges (all *p*_FDR_ > 0.05).

Between-group differences of edge strengths in the thalamocortical and thalamosubcortical network did not survive false discovery rate (FDR) correction (all *p*_FDR_ > 0.05).

### IDSCNs

IDSCNs were created using the *Network Template Perturbation* method to generate a unique SCN for each subject in the PTSD group. Overall PTSD severity, *DSM-5*-defined symptom clusters (re-experiencing, avoidance, negative mood/cognition, and hyperarousal symptoms), and comorbid depression severity were tested for correlations with the total number of altered edges per IDSCN, the number of altered edges per thalamic nucleus of each IDSCN, and the strength of individual network edges involving thalamic nuclei with heightened group-level centrality in PTSD subjects.

#### Total number of altered edges correlates with symptom severity.

The total number of altered edges in intrathalamic IDSCNs significantly positively correlated with depression severity (*r* = 0.107, *p* = 0.001). The total number of altered edges in thalamocortical IDSCNs showed a weak positive correlation with depression severity that did not reach statistical significance (*r* = 0.069, *p* = 0.054). The total number of altered edges in thalamosubcortical IDSCNs significantly positively correlated with avoidance symptoms (*r* = 0.093, *p* = 0.027).

In the intrathalamic network, the number of altered edges per thalamic nucleus was significantly correlated with depression severity in 41 of the 50 nuclei (all *p*_FDR_ < 0.05). In the thalamocortical network, a greater number of altered thalamocortical edges involving the right Pt nucleus significantly correlated with depression severity (*r* = 0.121, *p*_FDR_ = 0.038). All analyses were rerun using multiple linear regression while adjusting for sex, age, and age^2^ with consistent results.

#### Specific intrathalamic and thalamocortical edges correlate with symptom severity.

In the intrathalamic network, PTSD severity, symptom cluster severity, and depression severity did not significantly correlate with edge strength between 12 nuclei that exhibited significantly altered centrality in PTSD. However, expanding the analysis to include all 1,225 network edges revealed significant negative correlations between PTSD severity and edge strength of the right PuL with the bilateral AV (left: *r* = −0.132, *p*_FDR_ = 0.004; right: *r* = −0.108, *p*_FDR_ = 0.036), bilateral VA (left: *r* = −0.117, *p*_FDR_ = 0.018; right: *r* = −0.103, *p*_FDR_ = 0.049), left VAmc (*r* = −0.106, *p*_FDR_ = 0.038), left CL (*r* = −0.114, *p*_FDR_ = 0.019), and left Pc (*r* = −0.117, *p*_FDR_ = 0.018) nuclei, as well as between the right AV and right Pc (*r* = −0.102, *p*_FDR_ = 0.049) nuclei. As PTSD severity increased, the perturbation of SC from the control group shifted from positive to negative. Analyses were rerun using multiple linear regression while adjusting for sex, age, and age^2^ with consistent results.

Examining thalamocortical network connections among the 12 thalamic nuclei that exhibited significantly altered centrality in PTSD, and all cortical brain regions revealed hyperarousal symptoms to significantly correlate with edge strengths between the Re, CeM, LSg, and CM nuclei and cortical regions across the left hemisphere temporal cortex, lateral orbitofrontal cortex, and lateral occipital cortex (Table 2). Expanding the analysis to include all 3,400 network edges revealed additional significant correlations between the bilateral Pt and the left superior temporal gyrus (left Pt: *r* = −0.158, *p*_FDR_ = 0.046; right Pt: *r* = −0.161, *p*_FDR_ = 0.046) and between the right CeM nucleus and the left lateral occipital cortex (*r* = −0.177, *p*_FDR_ = 0.031; Supporting Information Table S4). Edges between the right Re nucleus and the left lateral occipital cortex and between the left Re and right CM nuclei and the left superior temporal gyrus survived FDR correction when accounting for all 3,400 network edges (*p*_FDR_ < 0.05). Analyses were rerun using multiple linear regression while adjusting for sex, age, and age^2^ with consistent results.

**Table 2.** Correlations between hyperarousal symptoms and thalamocortical edge strength across the IDSCNs among nuclei that exhibited significantly altered centrality in the PTSD networks

**Thalamic nucleus**	**Cortical region**	**Correlation**	***p***	***p*_FDR_**
Left Re	Left Superior Temporal	−0.171	1.83E−05	0.015
Right Re	Left Lateral Occipital	−0.163	4.70E−05	0.019
Right CM	Left Superior Temporal	−0.158	8.07E−05	0.022
Left CeM	Left Superior Temporal	−0.153	1.40E−04	0.028
Left LSg	Left Temporal Pole	0.144	3.11E−04	0.049
Left Re	Left Transverse Temporal	−0.141	4.18E−04	0.049
Right Re	Left Lateral Orbitofrontal	−0.140	4.76E−04	0.049
Left Re	Left Middle Temporal	−0.140	4.78E−04	0.049
Right CM	Right Insula	−0.135	7.93E−04	0.065
Right CM	Left Insula	−0.134	7.99E−04	0.065
Right LSg	Left Temporal Pole	0.132	9.53E−04	0.068
Right Re	Left Pars Orbitalis	−0.131	1.04E−03	0.068
Right Re	Left Superior Temporal	−0.130	1.17E−03	0.068
Left CeM	Left Lateral Occipital	−0.130	1.17E−03	0.068
Left Re	Right Inferior Temporal	−0.129	1.34E−03	0.070
Right Re	Left Transverse Temporal	−0.128	1.37E−03	0.070
Left CM	Left Superior Temporal	−0.122	2.30E−03	0.098
Left CeM	Right Insula	−0.122	2.33E−03	0.098
Right CM	Left Lateral Occipital	−0.122	2.36E−03	0.098
Right Re	Right Inferior Temporal	−0.122	2.40E−03	0.098

Edges with *p*_FDR_ < 0.1 are shown.

### Hierarchical Clustering of Thalamic Nuclei

Group-level SCNs were used to hierarchically cluster thalamic nuclei according to their pattern of intrathalamic SC_diff_ in PTSD using average-linkage clustering. A Kelley–Gardner–Sutcliffe (KGS) penalty function was used to determine the optimal number of clusters. The same procedure was performed to cluster thalamic nuclei based on their pattern of thalamocortical SC_diff_ in PTSD. Left and right hemisphere nuclei were clustered separately. Hierarchical clustering results were then compared between the intrathalamic and thalamocortical clusterings using cophenetic correlations.

**Figure 4.**
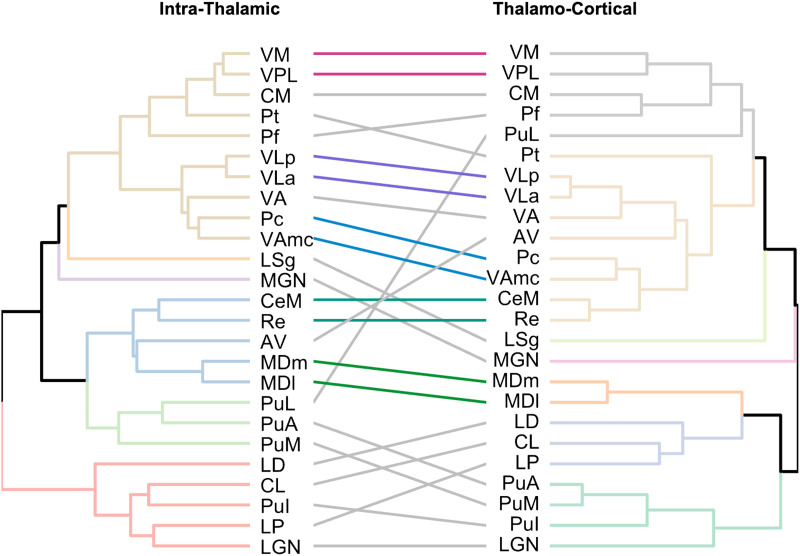
Left hemisphere thalamic nuclei clustering based on their pattern of altered intrathalamic and thalamocortical SC in PTSD. Colored connecting lines of the tanglegram represent pairs of nuclei that clustered together at the lowest level of the hierarchy in both network clusterings. Dendrogram branches are colored according to cluster membership.

For the left hemisphere, nuclei clustered into six and seven groups for the intrathalamic and thalamocortical networks, respectively. Left hemisphere clustering results across both networks displayed a cophenetic correlation of *r* = 0.52, indicating a strong similarity between the intrathalamic and thalamocortical clusterings. Figure 4 displays a tanglegram of the left hemisphere hierarchical clustering results to visualize the similarities and differences between clusterings.

For the right hemisphere, nuclei clustered into eight and six groups for the intrathalamic and thalamocortical networks, respectively. Right hemisphere clustering results across both networks displayed a cophenetic correlation of *r* = 0.21, indicating a moderate similarity for the right hemisphere clusterings, albeit considerably lower than the left hemisphere. Right hemisphere clustering results can be seen in Supporting Information Figure S1.

Notably, the Re and CeM nuclei clustered together bilaterally in both networks, and the CM and Pt nuclei clustered closely together in three of the four clusterings. Both the LSg and MGN clustered by themselves in both networks.

## DISCUSSION

We investigated how thalamic nuclei are affected in PTSD by mapping intrathalamic, thalamocortical, and thalamosubcortical network architecture with SC and individual differential SC analyses. At the global scale, higher graph strength between thalamic nuclei and between the thalamus and cortex was observed in PTSD, while graph strength between the thalamus and subcortex did not display significant group differences. Higher intrathalamic centrality was observed among the bilateral LSg, CM, and Re nuclei and the left MGN, VA, VPL, MDm, MDl, and CeM nuclei in PTSD. At the subject-level, greater PTSD severity was negatively correlated with intrathalamic SC involving the PuL nucleus. Comorbid depression severity was positively correlated with global and nodal alterations to the intrathalamic network, and avoidance symptoms positively correlated with global alterations to the thalamosubcortical network among PTSD subjects. Hyperarousal symptoms correlated with specific regional differences in the thalamocortical network involving midline limbic nuclei (Re, CeM, Pt), the CM nucleus, and the LSg nucleus with lateral occipital, temporal, and orbitofrontal cortices among PTSD subjects.

We found that comorbid depression severity was related to global alterations to the intrathalamic network and altered thalamocortical SC in the Pt nucleus among PTSD subjects. The Pt nucleus is a midline limbic nucleus with strong structural connections to medial prefrontal, entorhinal, and hippocampal regions (Vertes et al., 2015)—brain regions often implicated in depression symptomatology (Belleau et al., 2019; I. B. Kim & Park, 2021). Avoidance symptoms correlated with global alterations to the thalamosubcortical network in PTSD. Trait harm avoidance in healthy samples and PTSD avoidance symptoms have both been linked to the amygdala, hippocampus, and striatum (Crombie et al., 2021; Iidaka et al., 2006; Laricchiuta et al., 2014; Zhong et al., 2024). Our results highlight the possibility that altered communication between the Pt nucleus and the cortex may relate to greater depression symptoms among PTSD patients, while altered communication between the thalamus and other subcortical structures may relate to increased avoidance behaviors among PTSD patients.

Midline limbic nuclei (Re, CeM, MDm, MDl) displayed stronger SC with the VPL, LSg, MGN, and CM nuclei in PTSD. Prior research implicates the VPL and LSg nuclei in somatosensory and nociceptive processing, with strong projections to somatosensory and insular cortices (Jones, 1985; O’Reilly et al., 2021). The LSg nucleus, along with the MGN, is additionally strongly involved in auditory processing and displays dense projections to the temporal cortex (Jones, 1985). The CM nucleus contains strong projections to the striatum and motor cortex and functions to promote associative learning through updating action-outcome contingencies (Yamanaka et al., 2018). Our results suggest an altered structural relationship between the limbic thalamus and the somatomotor and auditory thalamus in PTSD. Further, PTSD severity negatively correlated with SC between the PuL, which responds to complex auditory and visual stimuli and projects strongly to the occipital and temporal cortices (Kaas & Lyon, 2007), and nuclei spanning the anterior (AV), ventral (VA, VAmc), and intralaminar (Pc, CL) subregions of the thalamus in PTSD. This further points to altered structural relationships involving the auditory thalamus in PTSD.

With regard to the cortex, hyperarousal symptoms negatively correlated with SC between the CM, Pt, Re, and CeM nuclei and the temporal cortex; the Re and CeM nuclei and the lateral occipital cortex; and the Re nucleus and the lateral orbitofrontal cortex, and the hyperarousal symptoms positively correlated with SC between the LSg and the temporal pole. These results complement the intrathalamic findings of increased SC between the limbic thalamus and nuclei that project to the temporal cortex, further suggesting a disrupted relationship between the limbic thalamus and temporal regions in PTSD.

Clustering analysis revealed intrathalamic and thalamocortical patterns of altered SC in PTSD to be moderately correlated, and we observed complimentary findings across the two network analyses. This suggests that altered intrathalamic covariance patterns may reflect patterns of altered thalamocortical covariance and can aid in the interpretation of thalamocortical findings. Moreover, clustering results showed that the Re and CeM nuclei, and similarly the CM and Pt nuclei, displayed highly similar patterns of altered intrathalamic and thalamocortical SC in PTSD. Based on consensus data across multiple mammalian species, the Re and caudal CeM nuclei are a part of a distinct cluster of midline thalamic nuclei involved in multisensory and affective processing, with evidence of direct projections to temporal and orbitofrontal regions (Van der Werf et al., 2002). Strong connections with subcortical structures and with the prefrontal cortex position these nuclei to modulate heteromodal information and integrate memory and contextual information with perceptual processes.

The occipital-temporal cortex is heavily involved in audiovisual processing (Degerman et al., 2007) and guides the selection of behavioral responses by corepresenting affective and sematic dimensions of perceptual stimuli (Abdel-Ghaffar et al., 2024). Additionally, anterior/superior temporal and lateral orbitofrontal cortices are highly interconnected heteromodal regions crucial for socio-emotional processing (Lieberman, 2007). For instance, the superior temporal cortex functions to identify biological motion, recognize emotion, and understand the intentions of others (Thye et al., 2018), while the lateral orbitofrontal cortex generates affective evaluations of multisensory percepts (Cunningham et al., 2011). Altered volume (De Bellis et al., 2002; Kunimatsu et al., 2020), cortical thickness (Nilsen et al., 2016), and functional activation (Kunimatsu et al., 2020) of the temporal cortex have been reported in PTSD. Our results may implicate thalamotemporal connectivity in the altered structural and functional properties reported in the temporal cortex in PTSD. We speculate that PTSD may be associated with a greater influence of sensory information from the LSg in the anterior temporal cortex, along with reduced integration of contextual information from the limbic thalamus and reduced associability updating carried out by the CM nucleus. This may lead to heightened sensory reactivity and weaker perceptual evaluative processing in lateral cortical regions, potentially resulting in greater hyperarousal symptoms among PTSD patients.

Similar patterns of thalamocortical connectivity changes have been noted in several other psychiatric conditions, and it has been hypothesized that these patterns of altered thalamocortical connectivity may reflect a transdiagnostic effect (Hwang et al., 2022). Indeed, previous investigation of SC in schizophrenia reported decreased SC between the whole thalamus and the ventrolateral prefrontal, superior temporal, and occipital cortices (Zhang et al., 2014). Investigation of individuals with major depressive disorder (MDD) showed stronger resting-state functional connectivity (rsFC) in MDD compared with controls between the midline thalamus and the temporal and somatosensory cortex (Brown et al., 2017). Similar findings have also been reported in bipolar disorder (Anticevic et al., 2014; Lu et al., 2023) and autism spectrum disorder (Nair et al., 2015). Finally, in PTSD, altered whole-thalamus rsFC to the somatomotor and ventrolateral prefrontal cortices has been reported (Jeon et al., 2020; Yin et al., 2011). The stark similarity between previous findings of thalamocortical SC and rsFC in other psychiatric conditions and the present results strongly suggests that aspects of our results may represent a general psychopathological trait rather than being specific to PTSD. Our results further support the hypotheses proposed by previous researchers (Brown et al., 2017; Hwang et al., 2022) that altered thalamocortical connectivity patterns reflect a transdiagnostic phenomenon. Using a detailed segmentation of the thalamus, we extend our understanding of which histologically defined nuclei may be involved in PTSD.

The observed alterations in SC may be driven by multiple neurobiological mechanisms. One possibility is that altered SC reflects differences in direct white matter projections between regions (Paquola et al., 2018; Yee et al., 2018). These findings may also arise from altered functional coupling facilitated by shared network engagement (Paquola et al., 2018), particularly for intrathalamic connections given that not all thalamic nuclei are directly connected. Such alterations may arise from stress-induced neuroplasticity (Deppermann et al., 2014), aberrant myelination (Chao et al., 2015), or compensatory functional reorganization (Cisler et al., 2014) in response to trauma. Future research will be needed to uncover the precise contributions of these mechanisms to altered thalamic SC in PTSD.

### Limitations and Strengths

Our study has several limitations. SC analysis examines how brain regions differentially covary with each other across groups, but it cannot explain whether changes in covariation are due to connectivity changes, alterations to a shared molecular environment, or some other confounding factor. Additionally, data on medication use and psychiatric comorbidities beyond MDD were not available. Strengths of the current study include aggregating data collected across multiple sites and using *ComBat-GAM* for scanner harmonization. *ComBat-GAM* is currently recognized as one of the best methods for removing site- or scanner-related variability from neuroimaging data (Sun et al., 2022). A large sample size and a detailed segmentation of the thalamus are additional strengths of the current study. By aggregating data from the largest data repository in the world for neuroimaging of PTSD subjects, we were able to substantially increase statistical power to detect differences across groups that may go undetected in single cohort studies. Using a probabilistic atlas of 25 thalamic nuclei in each hemisphere to obtain volumetric estimates of each nucleus, we constructed detailed networks of thalamic nuclei to uncover specific patterns of altered SC in PTSD.

### Conclusions

Thalamic involvement in a broad range of cognitive, affective, sensorimotor, and memory-related functions makes it an important structure to understand in relation to psychopathology. Altered intrathalamic, thalamocortical, and thalamosubcortical network properties in relation to PTSD suggest broad changes in thalamic functions posttrauma. Within the thalamus, limbic-projecting nuclei were found to more strongly covary with somatomotor and auditory nuclei in PTSD. Hyperarousal symptoms negatively correlated with SC between the limbic thalamus and lateral cortex. Differences in the relationship between affective and memory-related thalamic structures with multisensory cortical regions may help explain a range of psychopathological symptoms commonly seen in PTSD. Moderate clustering similarity and complementary findings across intrathalamic and thalamocortical networks highlight intrathalamic network architecture as a valuable source of information regarding the interpretation of thalamocortical structural connectivity patterns.

## METHODS

### Sample

Clinical and demographic information for subjects from the ENIGMA-PGC PTSD working group are shown in Table 3. The 2,784 subjects were assigned to a PTSD group (*n* = 1,306)—consisting of individuals who met either *DSM-IV* or *DSM-5* criteria for PTSD—or to a control group (*n* = 1,478; 86.98% trauma-exposed) who had neither a PTSD nor MDD diagnosis. Psychiatric diagnoses and symptom severity scores were determined at each site using either clinical diagnostic interviews or self-report symptom questionnaires (see Supporting Information Table S1). To allow comparison between symptom severity measures from different assessments, each subject's score was normalized to a value between 0 and 1, based on the minimum and maximum possible score for the relevant assessment. All study procedures were approved by local institutional review boards (IRBs), and all subjects provided written informed consent. Secondary data analysis was deemed exempt by the Duke University Medical Center IRB.

**Table 3.** Clinical and demographic data for PTSD and control subjects

	**PTSD**	**Controls**	***p***
*N*	1,306	1,478	
Age	40.46 (13.0)	42.7 (14.3)	0.056
Female %	44.70%	30.40%	<0.001
PTSD severity	0.51 (0.16)	0.11 (0.11)	<0.001
**PTSD symptom clusters**			
*N*	729	639	
Re-experiencing	0.47 (0.21)	0.09 (0.13)	<0.001
Avoidance	0.54 (0.25)	0.09 (0.17)	<0.001
Mood/cognition	0.41 (0.21)	0.04 (0.08)	<0.001
Hyperarousal	0.50 (0.18)	0.11 (0.13)	<0.001
**MDD**			
*N*	884	1,478	
MDD %	71.09%	0%	<0.001
MDD severity	0.43 (0.20)	0.10 (0.10)	<0.001
Alcohol use			
*N*	278	380	
Dependence/abuse %	23.02%	4.47%	<0.001
**CTQ**			
*N*	370	304	
CTQ total	63.46 (23.74)	38.40 (14.08)	<0.001
Emotional abuse	15.50 (6.53)	7.91 (3.94)	<0.001
Physical abuse	10.85 (5.57)	7.42 (3.58)	<0.001
Sexual abuse	12.39 (7.44)	6.58 (3.97)	<0.001
Emotional neglect	14.85 (5.94)	8.84 (4.24)	<0.001
Physical neglect	10.43 (4.64)	6.62 (2.52)	<0.001

Means are reported, with standard deviations in parentheses. *p* Values (uncorrected) of *t* tests for continuous variables and chi-squared tests for categorical variables are also reported. Symptom severity scores have been normalized across assessments to a value between 0 and 1. CTQ = Childhood Trauma Questionnaire.

### Data Preparation

T1-weighted brain MRI scans were shared by each site (see Supporting Information Table S2 for the scanning parameters of each site) and processed and segmented via the *SegmentThalamicNuclei* pipeline within FreeSurfer (Version 7.1.1) to segment the thalamus into 25 distinct nuclei per hemisphere. Volumetric estimates were obtained for each of the 25 left and 25 right hemisphere thalamic nuclei. Volumetric estimates for 34 cortical brain regions in each hemisphere from the Desikan–Killiany atlas were extracted. Subcortical brain volumes from six subcortical structures (caudate, putamen, pallidum, nucleus accumbens, amygdala, and hippocampus) in each hemisphere were extracted from FreeSurfer's automated segmentation. Subcortical data were not available for one site (*n* = 825).

Scans with statistical outlier volumes were removed to address concerns about missegmentation. Following previously established methods, scans with any brain region volume that exceeded ±2.698 *SD*s from the sample mean for that region were removed from statistical analysis (Huggins et al., 2024). This resulted in *n* = 550 subjects being removed from the intrathalamic network analysis, *n* = 991 from the thalamocortical network analysis, and *n* = 635 from the thalamosubcortical network analysis. As data were collected from multiple sites and MRI scanners, the *neuroHarmonize* package in python (Version 3.9.19) was used to adjust for site- and scanner-related variability in brain volumes. The *neuroHarmonize* package uses the *ComBat-GAM* algorithm that is based on an empirical Bayes framework (Pomponio et al., 2020), which adjusts for variability related to scanner type, while preserving variability related to variables of interest. The scanner-harmonized brain volumes were entered into a linear model to remove variability related to age, age^2^, sex, and intracranial volume. The residuals of this linear model were used as the input for the SC analysis. Regression was performed on all subjects as a single group. However, additional analyses were conducted using separate regression models for the PTSD and control groups to assess the potential impact of between-group differences in covariate effects, which produced consistent results across all analyses (data not shown).

### Creation of Thalamic SCNs

Pearson's correlation coefficients between the residuals of the linear model were calculated for each possible pairing of thalamic nuclei, independently for the PTSD and control groups, to generate an intrathalamic network. The result was a symmetrical 50 × 50 correlation matrix for each group. Network nodes refer to the 50 thalamic nuclei, and network edges were defined as the Pearson's correlation between the volumes of each pair of nodes. Similarly, to generate a thalamocortical network, the Pearson's correlation coefficients between the residuals of the linear model were calculated for each possible pairing of thalamic nuclei and cortical brain region. A thalamosubcortical network was generated following the same approach as for the thalamocortical network. Intrathalamic and intracortical network edges were removed from the thalamocortical network to assess differences in thalamocortical connections in PTSD (Figure 1D). Intrathalamic and intrasubcortical network edges were removed from the thalamosubcortical network to assess differences in thalamosubcortical connections in PTSD.

Thresholded weighted graphs for a range of thresholds were constructed to ensure biologically nonsignificant correlations were not unduly impacting results. The same thresholding procedure was applied to both groups to achieve unbiased comparisons in the strength of SC between groups rather than focusing primarily on topological differences. Thresholding started at *r* = 0 and increased in steps of 0.025 until the threshold reached the minimum wiring cost for one of the groups (Palaniyappan et al., 2019). The minimum wiring cost refers to the lowest edge density (number of edges / number of possible edges) at which the graph has no disconnected nodes, such that every node can be reached via some path from every other node. The minimum wiring cost was reached at thresholds above *r* = 0.40 for the intrathalamic network and *r* = 0.10 for the thalamocortical and thalamosubcortical networks.

### Graph Theory Measures

Graph theory measures were calculated in R (Version 4.4.1) using the *Braingraph* package (Watson, 2024). Statistical significance of group differences between measures was determined by running nonparametric permutation tests where the group labels were randomly shuffled while keeping group size constant. The proportion of permutations resulting in a group mean difference that equaled or exceeded the observed group difference constituted the uncorrected *p* value. Correction of *p* values for multiple comparisons used the Benjamini–Hochberg method with an FDR of *q* = 0.05 (Benjamini & Hochberg, 1995). The number of permutations used varied across measures depending on the sensitivity needed for the number of planned comparisons being corrected. For graph-level and node-level analyses, 5,000 permutations were run. For edge-level analyses, 100,000 permutations were run. All permutation tests and statistical analyses were conducted using R scripts. All code is available at https://github.com/njsteele01/Thalamic_SCN_analysis.git.

#### Global and nodal measures.

Global and nodal network measures were restricted to measures that can be readily applied to bipartite graphs, as our thalamocortical and thalamosubcortical networks exhibit a bipartite structure. Traditional network measures, such as clustering coefficient and certain centrality metrics, are not well suited for bipartite graphs because they rely on properties like triadic closure, which is inherently absent in bipartite networks. Instead, our analysis used graph strength and node strength centrality as measures of network connectivity.

Graph strength was calculated for the PTSD and control groups as the sum of connection weights across the network. Graph strength results in a single value per graph, so no correction for multiple comparisons was required. Node strength (i.e., weighted degree) centrality was calculated for the 50 thalamic nuclei in the intrathalamic, thalamocortical, and thalamosubcortical networks. Strength centrality reflects how influential or important a node is to the network. Nodes with high centrality are generally more strongly connected to other nodes and propagate information more easily to other parts of the network.

#### Edge strength.

In the intrathalamic network, differences in the strength of individual edges in PTSD compared with controls (SC_diff_) were examined between nuclei that displayed significantly altered centrality. The difference between the PTSD and control group SCN was calculated and then filtered to only include nodes that displayed significantly altered centrality in any of the examined networks. In the thalamocortical and thalamosubcortical networks, edges between thalamic nuclei with significantly altered centrality and all (sub)cortical brain regions were tested for between-group differences in edge strength. This was done to minimize the number of comparisons for multiple comparison correction. However, additional analyses examining all edges in each network are also reported.

### IDSCN Analysis

IDSCNs were created for each subject in the PTSD group using the *Template Network Perturbation* method (Liu et al., 2021). First, the control group SCN was created by finding the correlation between each brain region across *n* control subjects without a PTSD diagnosis (SCN*_n_*). PTSD subject *k* was then added to the control group, and a new network was generated, deemed the perturbed SCN (SCN_*n*+1_). Finally, the difference between the control network and the perturbed network was calculated (IDSCN*_k_*; Equation 1).

$${\text{IDSCN}}_{k}=SCN_{n+1}-{SCN}_{n}$$ (1)

Values of IDSCN*_k_* were then converted to *Z* scores (Equation 2). *Z* scores were further converted to *p* values and FDR-corrected for the number of edges in the network. Each IDSCN represents unique information about how a subject in the PTSD group changes the SC of the control network.

$$Z={\text{IDSCN}}_{k}/\left({\left(1-{SCN}_{n+1}\right)}^{2}/\left(n-1\right)\right)$$ (2)

The total number of altered edges per IDSCN, the number of altered edges per node in each IDSCN, and the strength of network edges between thalamic nuclei that displayed altered centrality in the group-level networks were correlated with overall PTSD severity, *DSM-5*-defined PTSD symptom cluster (criteria B, C, D, and E) severity, and comorbid depression severity across the IDSCNs. Symptom clusters refer to re-experiencing, avoidance, negative mood/cognition, and hyperarousal symptoms, respectively.

### Hierarchical Clustering of Thalamic Nuclei

Left and right hemisphere thalamic nuclei were separately hierarchically clustered according to their pattern of SC_diff_ across the group-level intrathalamic network and the group-level thalamocortical network. Thalamosubcortical patterns were not clustered due to a lack of significant network differences between groups. An agglomerative clustering algorithm was used to cluster thalamic nuclei. Average-linkage clustering was chosen over other algorithms because it produced the highest cophenetic correlation (Sokal & Rohlf, 1962) between the hierarchical clusterings and the original distance matrices, and it provided the highest cophenetic correlations between within-network left and right hemisphere clusterings. The optimal number of clusters was determined using a KGS penalty function with an alpha value set to one (Kelley et al., 1996). Results of the two clustering analyses were compared using cophenetic correlations to examine the similarity of intrathalamic and thalamocortical SC_diff_ in PTSD. The aim of this analysis was to reveal which thalamic nuclei may be similarly impacted in PTSD by clustering them close together and to test the degree of similarity between intrathalamic and thalamocortical patterns of SC_diff_ in PTSD.

## ACKNOWLEDGMENTS

Department of Defense Award Number W81XWH-12-2-0012; ENIGMA was also supported in part by National Institutes of Health U54 EB020403 from the Big Data to Knowledge (BD2K) program, R56AG058854, R01MH116147, R01MH111671, and P41 EB015922. R01MH111671, R01MH117601, R01AG059874, MJFF 14848. The study was supported by ZonMw, The Netherlands organization for Health Research and Development (40-00812-98-10041), and by a grant from the Academic Medical Center Research Council (110614) both awarded to M.O. The National Natural Science Foundation of China (No. U21A20364 and No. 31971020), the Key Project of the National Social Science Foundation of China (No. 20ZDA079), the Key Project of Research Base of Humanities and Social Sciences of Ministry of Education (No.16JJD190006), and the Scientific Foundation of Institute of Psychology, Chinese Academy of Sciences (No. E2CX4115CX). Funding from the SAMRC Unit on Risk & Resilience in Mental Disorders. R01MH105355-01A. NARSAD 27040; National Institute of Mental Health (NIMH) K01 MH118428-01; RO1 MH111671; VISN6 MIRECC. MH098212; MH071537; M01RR00039; UL1TR000454; HD071982; HD085850. Narsad Young Investigator. MH101380. Supported by a grant from the Ministry of Health of the Czech Republic, grant no. AZV NV18-7 04-00559. R21MH112956, R01MH119227, McLean Hospital Trauma Scholars Fund, Barlow Family Fund, Julia Kasaparian Fund for Neuroscience Research. K01MH118467; Julia Kasparian Fund for Neuroscience Research. R01MH113574. VA RR&D 1IK2RX000709. VA RR&D 1K1RX002325; 1K2RX002922. VA RR&D I01RX000622; CDMRP W81XWH-08–2–0038 to Dr. Sponheim. German Research Society (Deutsche Forschungsgemeinschaft, DFG; SFB/TRR 58: C06, C07). The Natural Science Foundation of Jiangsu Province (No. BK20221554) and the Foundation for the Social Development Project of Jiangsu (No. BE2022705). 1R01MH110483 and 1R21 MH098198. PHRC, Fondation Pierre Deniker and SFR FED4226 (to Prof El-Hage). Dana Foundation (to Dr. Nitschke); the University of Wisconsin Institute for Clinical and Translational Research (to Dr. Emma Seppala); a National Science Foundation Graduate Research Fellowship (to Dr. Grupe); the NIMH R01-MH043454 and T32-MH018931 (to Dr. Davidson); and a core grant to the Waisman Center from the National Institute of Child Health and Human Development (P30-HD003352). R01 MH106574. VA CSR&D 1IK2CX001680; VISN17 Center of Excellence Pilot funding. VA National Center for PTSD; The Beth K and Stuart Yudofsky Chair in the Neuropsychiatry of Military Post Traumatic Stress Syndrome.

## SUPPORTING INFORMATION

Supporting information for this article is available at https://doi.org/10.1162/NETN.a.535.

## AUTHOR CONTRIBUTIONS

Nick Steele: Conceptualization; Formal analysis; Methodology; Software; Validation; Visualization; Writing – original draft; Writing – review & editing. Ahmed Hussain: Data curation; Writing – review & editing. Delin Sun: Methodology; Supervision. Lexi Baird: Data curation. Courtney Russell: Data curation. Neda Jahanshad: Data curation; Writing – review & editing. Lauren Salminen: Data curation. Miranda Olff: Data curation. Jessie Frijling: Data curation. Dick Veltman: Data curation. Saskia Koch: Data curation. Laura Nawijn: Data curation. Mirjam van Zuiden: Data curation. Li Wang: Data curation. Ye Zhu: Data curation. Gen Li: Data curation. Dan Stein: Data curation. Jonathan Ipser: Data curation. Sheri Koopowitz: Data curation. Yuval Neria: Data curation. Xi Zhu: Data curation. Orren Ravid: Data curation. Sigal Zilcha-Mano: Data curation. Amit Lazarov: Data curation. Benjamin Suarez-Jimenez: Data curation. Ashley Huggins: Data curation. Jennifer Stevens: Data curation. Kerry Ressler: Data curation. Tanja Jovanovic: Data curation. Sanne van Rooij: Data curation. Negar Fani: Data curation. Emily Dennis: Data curation. David Tate: Data curation. David Cifu: Data curation. William Walker: Data curation. Elisabeth Wilde: Data curation. Ivan Rektor: Data curation. Pavel Říha: Data curation. Milissa Kaufman: Data curation. Lauren Lebois: Data curation. Justin Baker: Data curation. Anthony King: Data curation. Israel Liberzon: Data curation; Writing – review & editing. Mike Angstadt: Data curation. Nicholas Davenport: Data curation. Seth Disner: Data curation. Scott Sponheim: Data curation. Thomas Straube: Data curation. David Hofmann: Data curation. Guang Ming Lu: Data curation. Rongfeng Qi: Data curation. Amit Etkin: Data curation. Adi Maron-Katz: Data curation. Xin Wang: Data curation. Austin Kunch: Data curation. Hong Xie: Data curation. Yann Quidé: Data curation; Writing – review & editing. Wissam El-Hage: Data curation. Shmuel Lissek: Data curation. Hannah Berg: Data curation. Steven Bruce: Data curation. Josh Cisler: Data curation. Marisa Ross: Data curation. Ryan Herringa: Data curation. Daniel Grupe: Data curation. Jack Nitschke: Data curation. Richard Davidson: Data curation. Christine Larson: Data curation. Terri deRoon-Cassini: Data curation. Carissa Tomas: Data curation. Jacklynn Fitzgerald: Data curation. Brandee Feola: Data curation. Jennifer Blackford: Data curation. Bunmi Olatunji: Data curation. Geoffrey May: Data curation. Steven Nelson: Data curation. Evan Gordon: Data curation. Chadi Abdallah: Data curation. Ruth Lanius: Data curation. Maria Densmore: Data curation. Jean Théberge: Data curation. Richard Neufeld: Data curation. Paul Thompson: Data curation; Writing – review & editing. Rajendra Morey: Methodology; Supervision; Validation; Writing – review & editing.

## CONFLICT OF INTERESTS

LAML reports unpaid membership on the Scientific Committee for the International Society for the Study of Trauma and Dissociation (ISSTD), grant support from the National Institute of Mental Health, K01 MH118467, and spousal IP payments from Vanderbilt University for technology licensed to Acadia Pharmaceuticals unrelated to the present work. ISSTD and NIMH were not involved in the analysis or preparation of the manuscript. R.J.D. is the founder and president of the board of directors and serves on the board of directors for the nonprofit organization Healthy Minds Innovations, Inc. C.G.A. has served as a consultant, speaker, and/or an advisory board for Douglas Pharmaceutical, Aptinyx, FSV7, Lundbeck, Psilocybin Labs, Genentech, and Janssen. C.G.A. filed a patent for using mechanistic Target of Rapamycin (mTOR) inhibitors to augment the effects of antidepressants (filed on August 20, 2018). W.E.H.: Air Liquide, CHUGAI, EISAI, Jazz Pharmaceuticals, Janssen, Lundbeck, Otsuka, UCB, none related to this work.

## Supplementary Material



## TECHNICAL TERMS

SC: Structural covariance, a statistical association or pattern of interregional volumetric covariation.
SCN: Structural covariance network, a network representation of a set of brain regions that covaries with other regions within a sample or subsample.
IDSCN: Individual differential structural covariance network, a subject-specific network derived by quantifying the differential structural covariance of a subject's regional brain volumes relative to a reference group.
Graph strength: The sum of the weights of all edges in a network, which reflects the overall degree of connectedness of a network.
Node centrality: A measure quantifying the relative importance or influence a network node has on the rest of the network.
Hierarchical clustering: An unsupervised algorithm that builds a nested sequence of clusters by merging or splitting them based on a defined similarity or distance metric.
Tanglegram: A visualization technique to compare the relationship between two hierarchical clusterings.
