# Supplementary material for: Disrupted intrathalamic and thalamocortical structural covariance networks in posttraumatic stress disorder
Source: Netw Neurosci. 2026 Mar 20;10(1):244–66. doi: 10.1162/NETN.a.535 (PMC13008379; doi:10.1162/NETN.a.535)
Supplement: Supplementary file 1 [file netn-10-1-244-s001.pdf]

Supplementary Materials

Site	*N*	Age	Female %	PTSD	Controls	PTSD Severity	Diagnosis Tool	Severity Tool	Sample
ADNI	94	68.38 (4.38)	0.00	45	49	0.22 (0.20)	CAPS-IV	CAPS-IV	Military
AMC	68	39.50 (9.88)	48.53	35	33	0.27 (0.24)	CAPS-IV	CAPS-IV	Police
Beijing	64	48.78 (10.65)	57.14	39	25	0.38 (0.23)	PCL-5	PCL-5	Civilian
Capetown	74	26.88 (6.14)	100.00	1	73	-	MINI	-	-
Columbia	72	35.49 (12.66)	45.07	24	48	0.25 (0.21)	SCID	CAPS-5	Civilian
Duke	120	40.02 (10.51)	21.67	33	87	0.18 (0.24)	CAPS-IV, CAPS-5, SCID	CAPS-IV, CAPS-5, DTS	Military
Emory	51	39.92 (12.18)	100.00	14	37	0.21 (0.17)	CAPS-IV	CAPS-IV, MPSS	Civilian
Ghent	45	38.27 (11.68)	100.00	5	40	-	MINI	-	-
Groningen	30	38.27 (10.18)	100.00	30	0	0.51 (0.09)	CAPS-IV	CAPS-IV	Civilian
LIMBIC-CENC	825	40.56 (9.93)	13.68	322	503	0.33 (0.25)	PCL-5	PCL-5	Military
Masaryk	241	52.15 (17.83)	63.90	98	143	0.18 (0.15)	PCL-C	PCL-C	Civilian
Mclean Kaufman	51	35.25 (13.23)	100.00	51	0	0.64 (0.14)	CAPS-5	CAPS-5	Civilian
Michigan	38	30.21 (8.07)	0.00	25	13	0.39 (0.27)	CAPS-IV	CAPS-IV	Military, Civilian
Milwaukee	37	33.07 (11.05)	64.86	12	25	0.19 (0.17)	CAPS-5	CAPS-5	Civilian
Minn VA	114	32.27 (7.97)	2.63	67	47	0.33 (0.20)	CAPS-IV	CAPS-IV	Military
Munster	41	25.59 (6.45)	85.37	16	25	-	SCID	-	-
Nanjing	136	57.33 (5.83)	54.01	49	87	0.21 (0.13)	SCID	CAPS-IV	Civilian
Ontario	124	38.36 (12.97)	70.63	94	30	0.39 (0.24)	CAPS-IV, CAPS-5	CAPS-IV, CAPS-5	-
Stanford	125	35.15 (10.64)	48.41	93	32	0.37 (0.23)	CAPS-IV	CAPS-IV	Military, Civilian
Toledo	45	37.24 (11.72)	36.36	14	31	0.17 (0.21)	CAPS-IV	CAPS-IV	Military, Civilian
Tours	35	27.91 (9.21)	100.00	10	25	0.32 (0.15)	CAPS-IV	CAPS-IV	Civilian
UMN	52	43.62 (9.37)	9.43	11	41	0.14 (0.16)	CAPS-IV	CAPS-IV	Military
UMSL	58	32.69 (9.84)	100.00	58	0	-	CAPS-IV	-	-
UW Cisler	96	33.50 (8.46)	100.00	77	19	0.44 (0.26)	CAPS-5, SCID	PCL-5, PCL-C	Civilian
UW Grupe	28	31.25 (6.63)	14.81	15	13	0.28 (0.25)	CAPS-IV	CAPS-IV	Military
Vanderbilt	35	31.31 (4.91)	22.22	8	27	0.10 (0.14)	CAPS-5	CAPS-5	Military
Waco VA	42	40.60 (10.70)	7.14	32	10	0.56 (0.30)	PCL-5	PCL-5	Military
West Haven	43	35.35 (10.19)	11.63	28	15	0.37 (0.21)	CAPS-IV	CAPS-IV	Military
**Overall**	**2,784**	**40.71 (13.79)**	**42.26**	**1,306**	**1,478**	**0.31 (0.24)**	**-**	**-**	**-**

Table S1: Demographic data and psychiatric assessment tools by site. Means are reported with standard deviations in parentheses for age and PTSD severity. PTSD diagnosis and severity was determined at each site using varied diagnostic tools. Severity scores were harmonized across each site based on the minimum and maximum possible score for each assessment, resulting in a severity score ranging from zero to one.

Site	Scanner ID	Scanner	Field Strength	Head coil channels	Sequence	Voxel size (mm)	FOV (mm)	Orientation	Repetition time (ms)	Echo time (ms)	Flip angle (degrees)
ADNI	adni_s1	GE Discovery MR750w	3T	40	FSPGR	1x1x1.2	256x256	Sagittal	7652	3.1	11
	adni_s2	GE Discovery MR750	3T	8	SPGR	1x1x1.2	256x256	Sagittal	6984	2.85	11
	adni_s3	GE Signa HDxt	3T	8	SPGR	1x1x1.2	256x256	Sagittal	7340	3.04	11
	adni_s4	Siemens TIM Trio	3T	12	MPRAGE	1x1x1.2	256x256	Sagittal	2300	2.98	9
AMC	amc_s1	Philips Achieva	3T	32	FAST MPRAGE	1x1x1	240x188	Axial	8200	3.8	8
Beijing	beijing_s1	Philips Achieva	3T	8	EPI	1x1x1	220x220	Axial	8500	3.7	8
Capetown	cape_town_s1	Siemens Skyra	3T	4	MPRAGE	1x1x1.5	256x256	Sagittal	2530	1.69 / 3.55 / 5.41 / 7.27	7
	tygerberg_s1	Siemens Allegra	3T	4	MPRAGE	1x1x1.5	256x256	Sagittal	2000	1.53 / 3.21 / 4.89 / 6.57	20
Columbia	columbia_s1	GE Signa Excite	1.5T	8	MPRAGE	1x1x1.3	256x256	Axial	7250	3	7
Duke	duke_s1	GE Discovery MR750	3T	8	FSPGR BRAVO	.9375x.9375x1	240	Axial	3.22	8.148	12
	duke_s2	GE Discovery MR750	3T	8	FSPGR	.9375x.9375x1.9	256	Axial	7.84	2.98	12
	duke_s3	GE Discovery MR750	3T	8	FSPGR BRAVO	1x1x1	256	Axial	8.16	3.18	12
	duke_s4	GE LX Nvi	4T	8	Spin-echo co-planar	1x1x1.9	240	Axial	12	5.4	20
	duke_s5	GE Discovery MR750	3T	8	FSPGR BRAVO	1x1x1	256	Axial	8.16	3.18	12
	duke_s6	Philips Ingenia	3T	8	3D TFE SENSE	.9375x.9375x1	240	Axial	8.148	3.728	8
	duke_s7	GE Discovery MR750	3T	8	FSPGR BRAVO	.9375x.9375x1	240	Axial	8.208	3.22	12
	duke_s8	GE Signa EXCITE	3T	8	FSPGR BRAVO	.9375x.9375x1	240	Axial	8.148	3.22	12
Emory	emory_s1	Siemens TIM Trio	3T	12	MPRAGE	1x1x1	224x256	Axial	2600	3.02	8
Ghent	ghent_s1	Siemens TIM Trio	3T	32	MPRAGE	1x1x1	256x256	Transversal	2250	4.18	9
Groningen	groningen_s1	Siemens TIM Trio	3T	12	MPRAGE	1x1x1	256x256	Sagittal	1900	2.52	9
LIMBIC-CENC	cenc_s1	Philips Ingenia	3T		MPRAGE	1x1x1.2	256x256	Sagittal	6780	3.16	9
	cenc_s2	Siemens TIM Trio	3T		MPRAGE	1x1x1.2	240x256	Sagittal	2300	2.96	9
	cenc_s3	GE Signa HDxt	3T		SPGR	1x1x1.2	256x256	Sagittal	6280	2.78	11
	cenc_s4	Siemens Verio/Skyra Fit	3T		MPRAGE	1x1x1.2	240x256	Sagittal	2300	2.98	9
	cenc_s5	GE Discovery MR750	3T		SPGR	1x1x1.2	256x256	Sagittal	8156	3.18	11
	cenc_s6	Philips Achieva	3T		MPRAGE	1x1x1x1.2	256x256	Sagittal	6760	3.15	9
	cenc_s7	Siemens Prisma	3T		MPRAGE	0.8x0.8x0.8	300x320	Sagittal	2400	2.24	8
	cenc_s8	Siemens Prisma	3T		MPRAGE	0.8x0.8x0.8	300x320	Sagittal	2400	2.24	8
Masaryk	masaryk_s1	Siemens Prisma	3T	64	MPRAGE	1x1x1	224x224		2300	2.34	8
McLean Kaufman	mclean_kaufman_s1	Siemens TIM Trio	3T	32	MPRAGE	1.3x1.3x1.3	256x128	Sagittal	2530	3.31	7
Michigan	michigan_s1	GE Signa Excite	3T	8	IF-FSPGR	1x1x1	256x256	Axial	12300	5.3	9
Milwaukee	milwaukee_s1	GE Discovery MR750	3T	32	SPGR	1x0.9375x0.9375	240x240	Sagittal	9800	4.6	8
Minn VA	minn_va_s1	Siemens Tim Trio	3T	12	MPRAGE	1x1x1	256x256	Coronal	2530	3.7	7
Munster	munster_s1	Siemens Prisma	3T	32	MPRAGE	1x1x1	256x256	Sagittal	2130	2.28	8
Nanjing	nanjing_s1	GE Discovery MR750	3T	8	FSPGR BRAVO	1x1x1	240x240	Axial	8208	3.22	12
Ontario	ontario_s1	Siemens Biograph mMR	3T	32	MPRAGE	1x1x1	256x240x192	Axial	2300	2.98	9
Stanford	stanford_s1	GE Discovery MR750	3T	8	SPGR	1.5x0.9x1.1	220x220 / 240x240	Coronal	8000 / 8600	3.6 / 3.4	15
Toledo	toledo_s1	GE SignaX	3T	8	SPGR	1x1x1	256x256	Axial	8200	3.2	12
Tours	tours_s1	Siemens Verio	3T	12		1x1x1	256x256	Sagittal	1900	2.48	9
UMN	umn_s1	Siemens Prisma	3T	32		0.9x0.9x0.9					
UMSL	umsl_s1	Siemens TIM Trio	3T	12	MPRAGE	1x1x1	256x256	Sagittal	2400	3.13	8
UW Cisler	uw_cisler_s1	Philips Achieva X-Series	3T	32	MPRAGE	1x1x1	256x256	Sagittal	7500	3.7	9
	uw_cisler_s2	GE Discovery MR750	3T	8	MPRAGE	1x1x1	256x256	Axial	8200	3.2	12
UW Grupe	uw_grupe_s1	GE Discovery X750	3T	8	MPRAGE	1x1x1	256x256	Sagittal	1900	2.5	9
Vanderbilt	vanderbilt_s1	Philips Intera	3T	32		0.8x0.8x0.9	256x256	Sagittal	9000	4.6	9
Waco VA	waco_va_s1	Philips Achieva	3T	16	MPRAGE	0.9x0.9x0.9	256x256	Sagittal	7256	2.77	12
West Haven	west_haven_va_s1	Siemens TIM Trio	3T	32	MPRAGE	1x1x1	256x256	Sagittal	2530	2.71	7

Table S2: Structural MRI scanning parameters of each site. Sites that used multiple MRI scanners have each scanner's acquisition parameters listed.

Threshold	PTSD	Controls	SC_{diff}	*p*
Intra-Thalamic				
0	858.433	783.351	75.082	0.013
0.025	858.331	783.228	75.103	0.013
0.05	858.030	782.628	75.401	0.013
0.075	857.011	780.343	76.668	0.013
0.1	853.817	776.820	76.997	0.015
0.125	849.052	770.427	78.625	0.016
0.15	839.947	758.222	81.725	0.018
0.175	826.552	737.816	88.737	0.020
0.2	806.173	706.349	99.824	0.015
0.225	772.314	663.022	109.292	0.013
0.25	731.223	620.649	110.574	0.018
0.275	684.870	569.805	115.065	0.010
0.3	628.204	523.277	104.927	0.012
0.325	578.794	478.362	100.432	0.011
0.35	530.844	429.903	100.941	0.005
0.375	481.553	389.317	92.237	0.008
0.4	428.919	350.773	78.145	0.011
Thalamo-Cortical				
0	541.573	394.394	147.180	0.041
0.025	533.142	380.861	152.281	0.042
0.05	496.627	323.128	173.499	0.037
0.075	418.254	229.797	188.457	0.028
0.1	293.027	130.544	162.483	0.032
Thalamo-Subcortical				
0	206.517	216.696	-10.179	0.607
0.025	206.294	216.616	-10.323	0.602
0.05	205.360	215.397	-10.038	0.617
0.075	203.047	212.218	-9.172	0.661
0.1	197.863	205.308	-7.445	0.743

Table S3: Graph strength of the intra-thalamic, thalamo-cortical, and thalamo-subcortical networks for PTSD and controls.

Nucleus 1	Nucleus 2	PTSD	Controls	SC_{diff}	p	p_{FDR}
Right LSg	Left Re	0.294	0.182	0.113	0.002	0.028
Left VPL	Right Re	0.255	0.145	0.110	0.003	0.028
Right LSg	Right Re	0.293	0.184	0.108	0.003	0.028
Right CM	Left CeM	0.441	0.333	0.108	0.001	0.028
Right LSg	Left CeM	0.260	0.154	0.106	0.004	0.028
Left CM	Right Re	0.385	0.281	0.104	0.003	0.028
Right CM	Left Re	0.374	0.276	0.098	0.004	0.028
Left CM	Left MDl	0.442	0.344	0.098	0.003	0.028
Right CM	Left MDl	0.372	0.275	0.098	0.005	0.029
Left VPL	Left MDl	0.445	0.349	0.096	0.004	0.028
Right CM	Left MDm	0.420	0.324	0.096	0.004	0.028
Left CM	Left MDm	0.497	0.402	0.096	0.002	0.028
Left VPL	Left Re	0.275	0.180	0.095	0.012	0.048
Left LSg	Right CM	0.334	0.239	0.094	0.007	0.037
Left CM	Left Re	0.423	0.332	0.091	0.010	0.045
Left VA	Right CM	0.461	0.371	0.090	0.007	0.037
Left MGN	Right LSg	0.481	0.391	0.090	0.004	0.028
Right CM	Right Re	0.402	0.313	0.089	0.009	0.042
Left LSg	Left CM	0.375	0.288	0.087	0.011	0.046

Table S4: Intra-thalamic network edges with significantly greater strength in PTSD compared to controls among nuclei with significantly altered centrality in the PTSD networks.

Thalamic Nucleus	Cortical Region	Correlation	p	p_{FDR}
Right CeM	Left Lateral Occipital	-0.177	9.98e-06	0.031
Left Re	Left Superior Temporal	-0.171	1.83e-05	0.031
Right Re	Left Lateral Occipital	-0.163	4.70e-05	0.046
Right Pt	Left Superior Temporal	-0.161	5.78e-05	0.046
Left Pt	Left Superior Temporal	-0.158	7.65e-05	0.046
Right CM	Left Superior Temporal	-0.158	8.07e-05	0.046
Left CeM	Left Superior Temporal	-0.153	1.40e-04	0.068
Right Pt	Left Insula	-0.149	1.96e-04	0.083

Table S5: Correlations between hyperarousal symptoms and thalamo-cortical edge strength across the IDSCNs when including all 3,400 network edges in the analysis. Edges with p_{FDR}<0.1 are shown.

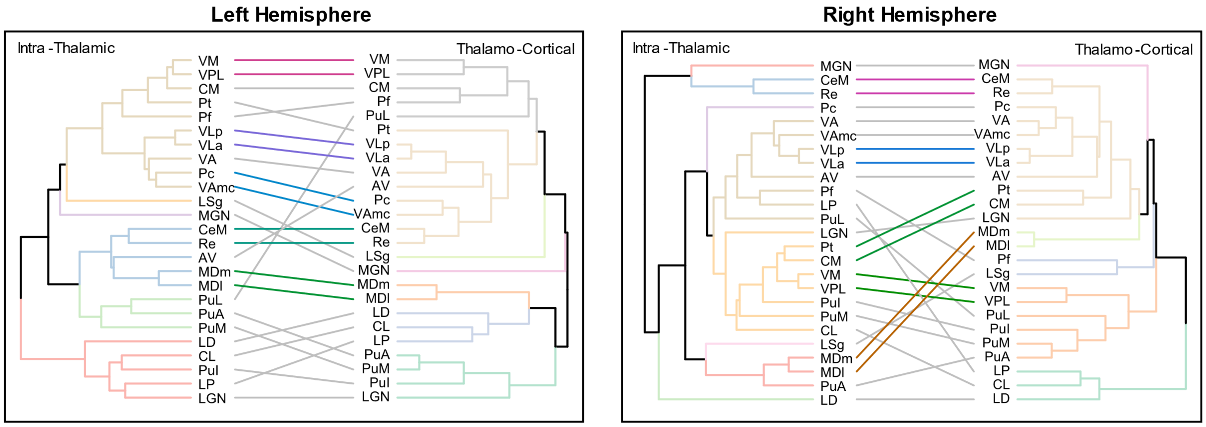

Figure S1: Tanglegram of left and right hemisphere clusterings for the intra-thalamic and thalamo-cortical networks.
